# Global research trends on the aryl hydrocarbon receptor in atopic dermatitis: a bibliometric and visualized analysis

**DOI:** 10.3389/fmed.2026.1840065

**Published:** 2026-06-24

**Authors:** Zhiquan Li, Chenwei Deng, Bingxuan Lu, Foling Chen, Xiyuan Cao, Jiwen Fan, Yu Song

**Affiliations:** 1 Clinical Medical College, Jiangxi University of Chinese Medicine, Nanchang, Jiangxi, China; 2 Longhua Hospital, Shanghai University of Traditional Chinese Medicine, Shanghai, China

**Keywords:** AHR, atopic dermatitis, bibliometrics, skin barrier, targeted therapy, visual analysis

## Abstract

**Background:**

Atopic dermatitis (AD) is a chronic inflammatory skin disease characterized by recurrent eczematous lesions and intense pruritus. The aryl hydrocarbon receptor (AHR), a ligand-activated transcription factor, plays a pivotal role in epidermal barrier differentiation, immune regulation, environmental responses, and microbiota–metabolite interactions, and has emerged as a potential therapeutic target with the clinical development of AHR agonists. However, a systematic bibliometric overview of the knowledge structure and evolving trends in this field is still lacking.

**Methods:**

After screening, deduplication, and standardization, English-language publications on AD and AHR published between 2005 and 2025 were retrieved from the Web of Science Core Collection (WoSCC) and Scopus. VOSviewer, CiteSpace, and Scimago Graphica were then used to visualize publication trends, collaboration networks, journal distribution, co-cited references, and keyword evolution.

**Results:**

A total of 306 publications were included, comprising 168 original articles and 138 reviews. Research on AHR in AD began in 2005 and entered a phase of rapid growth after 2020. The studies involved 49 countries/regions, with the United States leading in both publication output and international collaboration, followed by Japan, China, South Korea, and Germany as major contributors. Journal and co-citation analyses indicated that this field is characterized by the intersection of dermatology and basic immunology. Keyword clustering highlighted four major themes: immune and inflammatory regulation, skin barrier function and environmental exposure, the microbiota–metabolism–comorbidity axis, and AHR-targeted therapy and clinical evaluation. Overall, the research focus has gradually expanded from basic mechanisms to microbiota-targeted regulation and clinical translation.

**Conclusion:**

This study systematically mapped the research landscape and developmental trends of AHR in AD over the past two decades, revealing a clear shift from investigations centered on its role as a classical environmental sensor to multidimensional mechanistic studies involving immune regulation, skin barrier function, and the interplay among the microbiota, metabolism, and comorbidities. These findings provide clinicians and researchers with a rapid overview of the academic frontiers in this field and offer a useful reference for the development of AHR-targeted therapeutic strategies and the identification of future research directions.

## 1 Introduction

Atopic dermatitis (AD) is a chronic, relapsing inflammatory skin disorder and one of the most common chronic inflammatory dermatoses worldwide, clinically characterized by recurrent eczematous lesions and intense pruritus. It typically begins in infancy or early childhood, and in some patients persists into adulthood (1). AD is highly prevalent and follows a chronic, relapsing course, imposing a substantial disease burden on patients, including reduced quality of life, sleep disturbance, anxiety, depression, and high medical costs (2). The pathogenesis of AD involves multifactorial interactions among epidermal barrier dysfunction, immune dysregulation, and alterations in the skin microbiome (3, 4). Genetic susceptibility, together with environmental exposures such as pollution, climate, and microbial contact, collectively contributes to the onset and progression of the disease (5). Therefore, elucidating the key regulatory molecules involved in skin barrier homeostasis, immune-inflammatory responses, and environmental sensing is essential for deepening our understanding of AD pathogenesis and advancing the development of targeted therapies.

The aryl hydrocarbon receptor (AHR) is a ligand-dependent transcription factor widely expressed in epidermal keratinocytes that senses a broad range of endogenous and exogenous small-molecule signals (6). AHR initially attracted attention for mediating the toxic effects of environmental pollutants such as dioxins (7). However, it also plays a critical role in maintaining skin homeostasis. Extensive studies have demonstrated the multifaceted involvement of AHR in AD, with in-depth investigations spanning epidermal homeostasis, barrier repair, oxidative stress, the effects of environmental pollution, the microbiome, and tryptophan metabolism (8–10). Therefore, AHR has transcended its classical role as an environmental sensor and emerged as a key node in elucidating the pathogenesis of AD, owing to its critical functions in maintaining skin homeostasis and regulating immune responses, while also showing promise as a potential therapeutic target.

Meanwhile, the clinical development of AHR agonists, exemplified by tapinarof, has further advanced the field from mechanistic exploration to therapeutic translation. Recent clinical reviews and phase III randomized controlled trials have shown that AHR has emerged as a therapeutic target with tangible interventional potential (11, 12). However, the existing literature remains fragmented across multiple disciplines, including dermatology, allergy and immunology, environmental toxicology, microbiology, and pharmaceutical research. This lack of systematic synthesis makes it difficult to comprehensively define the knowledge base, identify key contributors, map collaborative networks, and trace the evolution of research hotspots. Therefore, a systematic analysis of the research hotspots and development trends of AHR in AD is particularly warranted.

Bibliometrics applies statistical methods to the study of scholarly publishing. By analyzing data such as authorship, research topics, and citation relationships, it characterizes publication trends within a field and reveals connections among the literature (13). This approach can delineate the research structure and evolutionary patterns of a specific field from multiple perspectives, enabling researchers to gain a comprehensive understanding of its current landscape, identify research hotspots, and anticipate future directions. However, a systematic bibliometric analysis of AHR in AD research is still lacking. Therefore, based on the WoSCC and Scopus databases, the present study systematically reviews AHR-related research in AD from multiple dimensions, including publication trends, collaboration among countries and institutions, author and journal distributions, the co-citation knowledge base, keyword clustering, and the evolution of emerging fronts. This study aims to clarify the current research landscape, key contributors, major topics, and future directions in this field, thereby providing a reference for subsequent mechanistic investigations and clinical translation.

## 2 Materials and methods

### 2.1 Data source and search strategy

High-quality bibliometric analyses depend critically on data-source selection, which directly affects the objectivity and comprehensiveness of the findings. WoSCC is widely recognized for its rigorous journal selection criteria and reliable citation-tracking system, offering distinct advantages in indexing high-quality literature. Scopus, by contrast, provides an effective complement through its broader disciplinary coverage and stronger capacity for cross-disciplinary citation analysis. Because any single database is inherently subject to indexing bias, combining these two sources enables a balance between the depth of WoSCC and the breadth of Scopus, thereby reducing the risk of retrieval omissions. This strategy provided a more representative literature base for the present study and offered a methodological foundation for identifying changes in the global research landscape, interdisciplinary trends, and emerging academic frontiers.

The literature search and data collection were completed in WoSCC and Scopus on February 4, 2026, with the search period restricted to January 1, 2005, through December 31, 2025. To ensure comprehensive and accurate retrieval, search terms were developed based on a preliminary literature review, and publication titles, abstracts, and keywords were used as the core search fields for initial screening. AD-related terms included (“atopic dermatitis” OR “atopic eczema” OR “atopic neurodermatitis” OR “atopic dermatit*” OR “atopic eczem*”). AHR-related terms included (“aryl hydrocarbon receptor*” OR “aromatic hydrocarbon receptor*” OR “Ah receptor*” OR “dioxin receptor*”). The detailed search strategy is provided in Supplementary Table 1. Because 2025 was the final year included in the search window and database indexing delays may still have affected records available at the time of data collection, records from 2025 were examined separately. This verification included the number of records retrieved from each database, the number excluded because of Early Access status or related reasons, the number remaining after merging and deduplication, and the final number included in the analysis. The corresponding details are provided in Supplementary Table 2.

### 2.2 Inclusion and exclusion criteria

**FIGURE 1:**
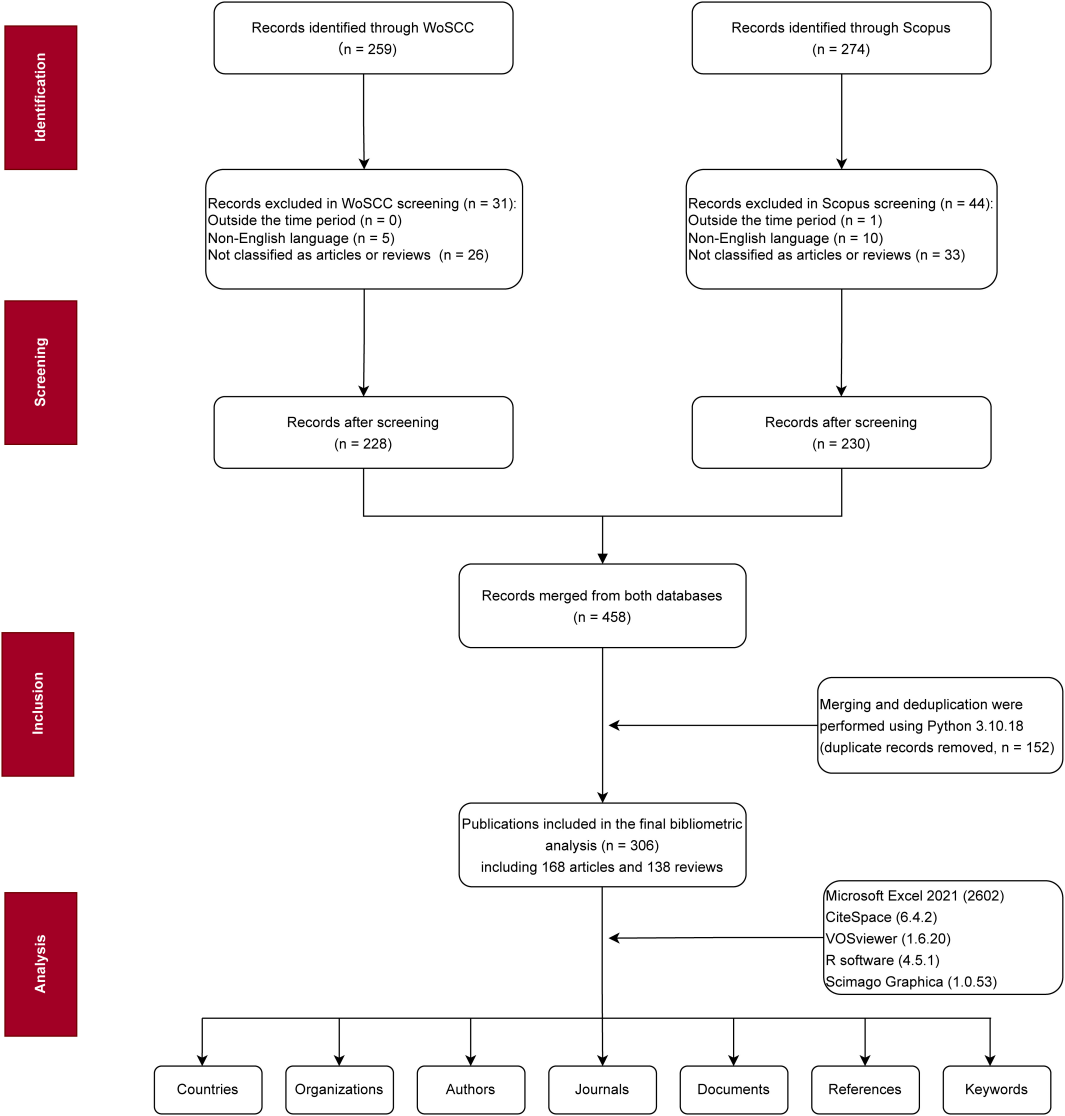
Flow diagram of the literature search and screening process.

Clear inclusion and exclusion criteria were established for this study. Literature screening was conducted independently by two researchers, and any disagreements were resolved through consultation with a third reviewer. The inclusion criteria were as follows: publications related to AD and AHR, published in English with complete bibliographic information, including clinical trials, *in vivo* studies, *in vitro* studies, public database analyses, and reviews. The exclusion criteria comprised Early Access articles, editorial materials, articles in press, book series, book chapters, conference abstracts, letters, duplicate publications, and retracted papers. After cross-database retrieval, Python was used to merge and deduplicate records from WoSCC and Scopus. Following this screening process, 306 publications were ultimately included for subsequent analysis, comprising 168 original articles and 138 reviews (Figure 1).

### 2.3 Data analysis

WoSCC records were exported as plain-text files with full records and cited references, whereas Scopus records were exported as CSV files using the same criteria. In the Python 3.10.18 environment, the Scopus data were converted to the WoSCC plain-text format and then merged with the WoSCC data, followed by deduplication to generate a unified TXT-format dataset. The data were subsequently preprocessed and standardized by removing special characters and redundant spaces. Keywords were normalized; for example, “atopic-dermatitis” and “dermatitis, atopic” were unified as “atopic dermatitis,” while “aromatic hydrocarbon receptor,” “receptors, aryl hydrocarbon,” and “AHR” were standardized as “aryl hydrocarbon receptor.” The country/region field was standardized according to the author affiliation addresses recorded in the source databases to ensure consistency in the bibliometric analysis and compliance with journal conventions for country/region nomenclature. After standardization, bibliographic metadata were reformatted using the data import/export function of CiteSpace to meet the requirements of subsequent analyses. The normalized dataset was then incorporated into a structured framework developed by two researchers, from which information on publication year, country/region, institution, journal, author, cited references, and keywords was extracted.

This study employed bibliometric visualization techniques to analyze research hotspots, knowledge structure, and evolutionary trends in the field of AD and AHR. For publication output analysis, data generated by CiteSpace were imported into Excel to generate a line chart of annual publication trends. Geographic visualization of countries/regions was based on VOSviewer outputs, which were converted into GML format and then mapped using Scimago Graphica. Author collaboration networks, institutional distribution, journal distribution, and keyword co-occurrence were analyzed using VOSviewer, with journal and author data additionally exported as CSV files for further processing. In VOSviewer analyses, link strength denotes the weight of the connection between two nodes, whereas total link strength (TLS) represents the sum of the link strengths between a given node and all other nodes within a given network. Country/region and institutional analyses were both based on co-authorship networks, and the collaboration TLS values reported in Tables 1, 3 reflect the overall co-authorship linkage of each node within the respective network; as these values were derived from separate network constructions, they are not directly comparable across tables. In journal analyses, the meaning of TLS depends on the type of network constructed: in citation networks, it represents the sum of citation link strengths between a given journal and other journals, whereas in co-citation networks, it represents the sum of co-citation link strengths. Burst keyword detection, keyword timeline analysis, and co-citation analysis were all performed in CiteSpace. The software versions, parameter settings, and screening thresholds used for each analysis are detailed in Supplementary Table 3.

**TABLE 1:** Top 10 countries/regions ranked by total publication output, 2005–2025.

Country/Region	Publications	Collaboration TLS	Total citations	Average citations per article
United States	88	59	4833	54.9205
Japan	50	10	3088	61.7600
China	48	7	1966	40.9583
South Korea	38	19	1024	26.9474
Germany	31	31	2460	79.3548
Canada	17	16	565	33.2353
United Kingdom	16	15	583	36.4375
Italy	13	19	511	39.3077
Switzerland	12	23	828	69.0000
Netherlands	10	11	697	69.7000

Collaboration TLS denotes the total link strength calculated by VOSviewer within the country/region co-authorship network.

**TABLE 2:** Top 10 most prolific authors and top 10 most highly co-cited authors in AD and AHR research, 2005–2025.

Rank	Author	Documents	Citations	Co-citations authors	Co-citations
1	Tsuji, G.	23	1257	Furue, M.	293
2	Furue, M.	22	1770	Bissonette, R.	164
3	Nakahara, T.	18	870	van den Bogaard, E. H.	132
4	Hashimoto-Hachiya, A.	12	716	Tsuji, G.	117
5	Rubenstein, D. S.	11	468	Simpson, E. L.	116
6	Tallman, A. M.	11	474	Guttman-Yassky, E.	115
7	Takemura, M.	9	296	Paller, A. S.	106
8	Kido-Nakahara, M.	8	496	Silverberg, J. I.	100
9	Gold, L. S.	8	404	Smith, H.	87
10	van den Bogaard, E. H.	8	681	Esser, C.	84

**TABLE 3:** Top 10 institutions in AD and AHR research ranked by publication output.

Rank	Institution	Location	Publications	Collaboration TLS	Total citations	Average citations per paper
1	Kyushu University	Japan	31	15	1863	60.10
2	Dermavant Sciences, Inc.	United States	13	38	489	37.62
3	Henry Ford Health System	United States	10	33	407	40.70
4	Northwestern University	United States	10	20	781	78.10
5	Icahn School of Medicine at Mount Sinai	United States	9	32	421	46.78
6	George Washington University	United States	8	32	207	25.88
7	Kyushu University Hospital	Japan	8	8	379	47.38
8	Radboud University Nijmegen	Netherlands	8	5	672	84.00
9	Hallym University	South Korea	8	3	219	27.38
10	Kyung Hee University	South Korea	7	9	185	26.43

Collaboration TLS denotes the total link strength calculated by VOSviewer within the institutional co-authorship network.

## 3 Results

### 3.1 Annual publication trends

**FIGURE 2:**
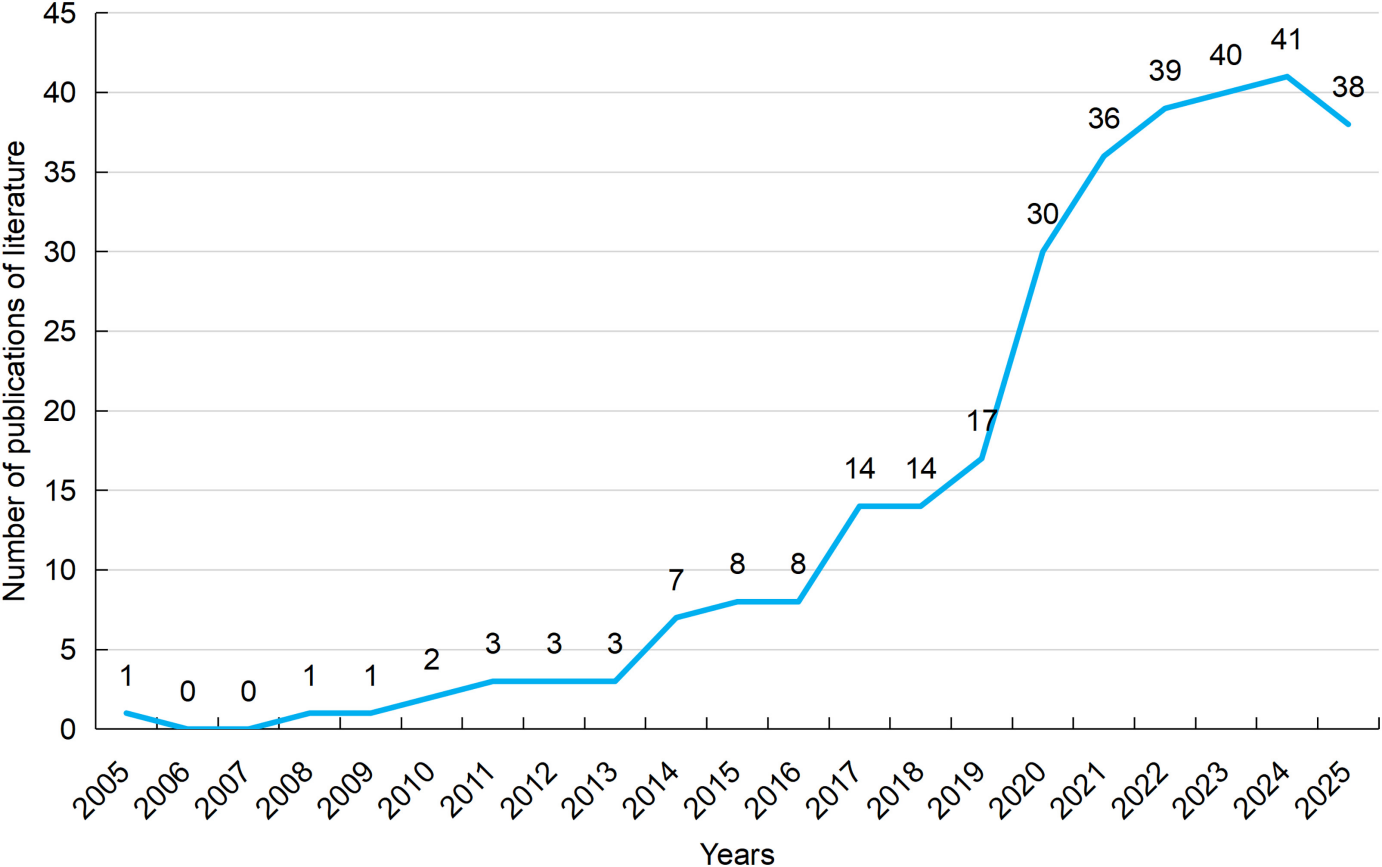
Annual publication trends in AHR-related research on AD from 2005 to 2025.

Figure 2 illustrates the annual publication output in this field over the past two decades. The earliest relevant publication appeared in 2005. From 2005 to 2013, annual output remained low (*n* ≤ 3), indicating that research was still in its early stage. Publications increased gradually between 2014 and 2019, followed by a marked surge after 2020, with an annual average of at least 30 publications and a peak in 2024 (*n* = 41). Notably, because database coverage and indexing of 2025 publications may remain incomplete, publication data for that year should not be used directly to assess full-year output or infer temporal trends. Overall, annual publication patterns indicate that AHR-related research in AD has expanded steadily over the past two decades, with a particularly marked increase in attention after 2020.

### 3.2 International collaboration network

**FIGURE 3:**
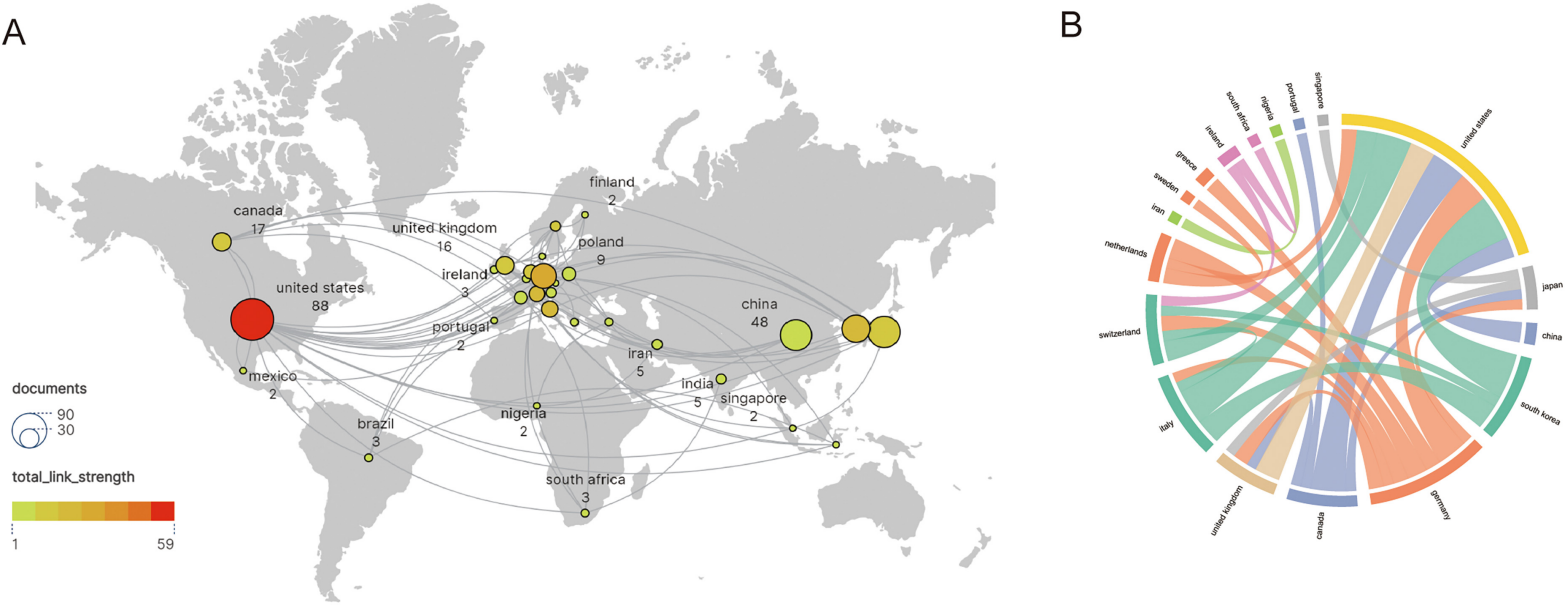
Visual analysis of AD and AHR research by country/region. **(A)** Global geographic distribution map. **(B)** Global collaboration chord diagram, in which node size represents publication output and line thickness indicates collaboration TLS.

Over the past two decades, scholars from 49 countries and regions have contributed to research on AD and AHR. Figure 3A illustrates the global distribution of publications, with the United States producing the largest number of articles (*n* = 88), followed by Japan (*n* = 50), China (*n* = 48), and South Korea (*n* = 38). The chord diagram in Figure 3B depicts international collaborations, showing that the United States had the highest collaboration TLS in the country/region co-authorship network (TLS = 59), while Germany (TLS = 31) and Switzerland (TLS = 23) also served as major collaboration hubs. Table 1 presents the top 10 most productive countries/regions, together with their collaboration TLS, total citations, and average citations per article. In terms of total citations, the United States ranked first (4,833), followed by Japan (3,088) and Germany (2,460). For average citations per article, Germany led with 79.35, indicating a particularly strong per-publication impact, while the Netherlands (69.70), Switzerland (69.00), and Japan (61.76) also showed outstanding performance. The geographic and citation distributions of these publications indicate that research at the intersection of AD and AHR has attracted substantial global attention and achieved considerable academic impact.

### 3.3 Author and institutional collaboration networks

**FIGURE 4:**
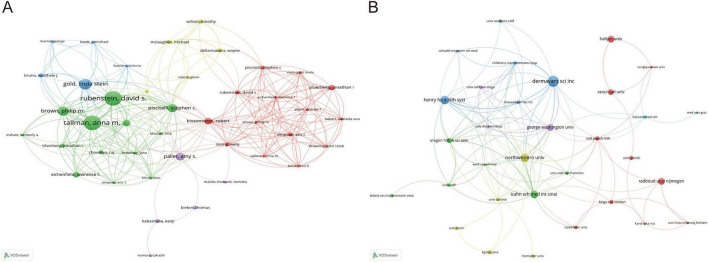
Visual analysis of authors and institutions in AD and AHR research: **(A)** author collaboration network; **(B)** institutional collaboration network.

Over the past 20 years, 1,472 authors from 541 institutions worldwide have contributed to publications on AD and AHR. The author collaboration network (Figure 4A) identified five highly active core research teams in this field, led by David S. Rubenstein, Linda Stein Gold, Robert Bissonette, Amy S. Paller, and Timothy Wilson, respectively, reflecting a relatively stable pattern of collaboration. The institutional collaboration network (Figure 4B) further showed that Dermavant Sciences, Inc., Northwestern University, Icahn School of Medicine at Mount Sinai, and George Washington University were particularly prominent in cross-institutional cooperation. These institutions established close partnerships with multiple research organizations and, to some extent, have driven the research direction and academic progress in this field.

Table 2 summarizes the leading productive authors and the most highly co-cited authors in AD and AHR research. In terms of publication output, Tsuji, G. ranked first (*n* = 23), followed by Furue, M. (*n* = 22) and Nakahara, T. (*n* = 18), together representing the most active research group in this field. Notably, Furue, M. received 1,770 total citations, far exceeding Tsuji, G. (*n* = 1,257) and Nakahara, T. (*n* = 870), highlighting the broad recognition and influence of his work within the field. Co-citation analysis further showed that Furue, M. also ranked first in co-citation frequency (*n* = 293), followed by Bissonette, R. (*n* = 164) and van den Bogaard, E. H. (*n* = 132). These findings suggest that the work of these scholars has become a major intellectual foundation in this field, exerting a sustained influence on knowledge accumulation and the shaping of research directions in AD and AHR research.

As shown in Table 3, Kyushu University (Japan) ranked first in both number of publications (*n* = 31) and total citations (*n* = 1,863). In terms of collaboration TLS in the institutional co-authorship network, Dermavant Sciences, Inc. ranked highest (TLS = 38), followed by Henry Ford Health System (TLS = 33), and Icahn School of Medicine at Mount Sinai and George Washington University (both TLS = 32), highlighting the prominent role of U.S. institutions in cross-institutional collaboration. Regarding average citations per publication, Radboud University Nijmegen (*n* = 84.00) and Northwestern University (*n* = 78.10) showed outstanding performance, indicating that their individual studies attracted substantial academic attention. Together, these institutions represent the core research forces in the field of AD and AHR from multiple perspectives.

### 3.4 Journal analysis

**FIGURE 5:**
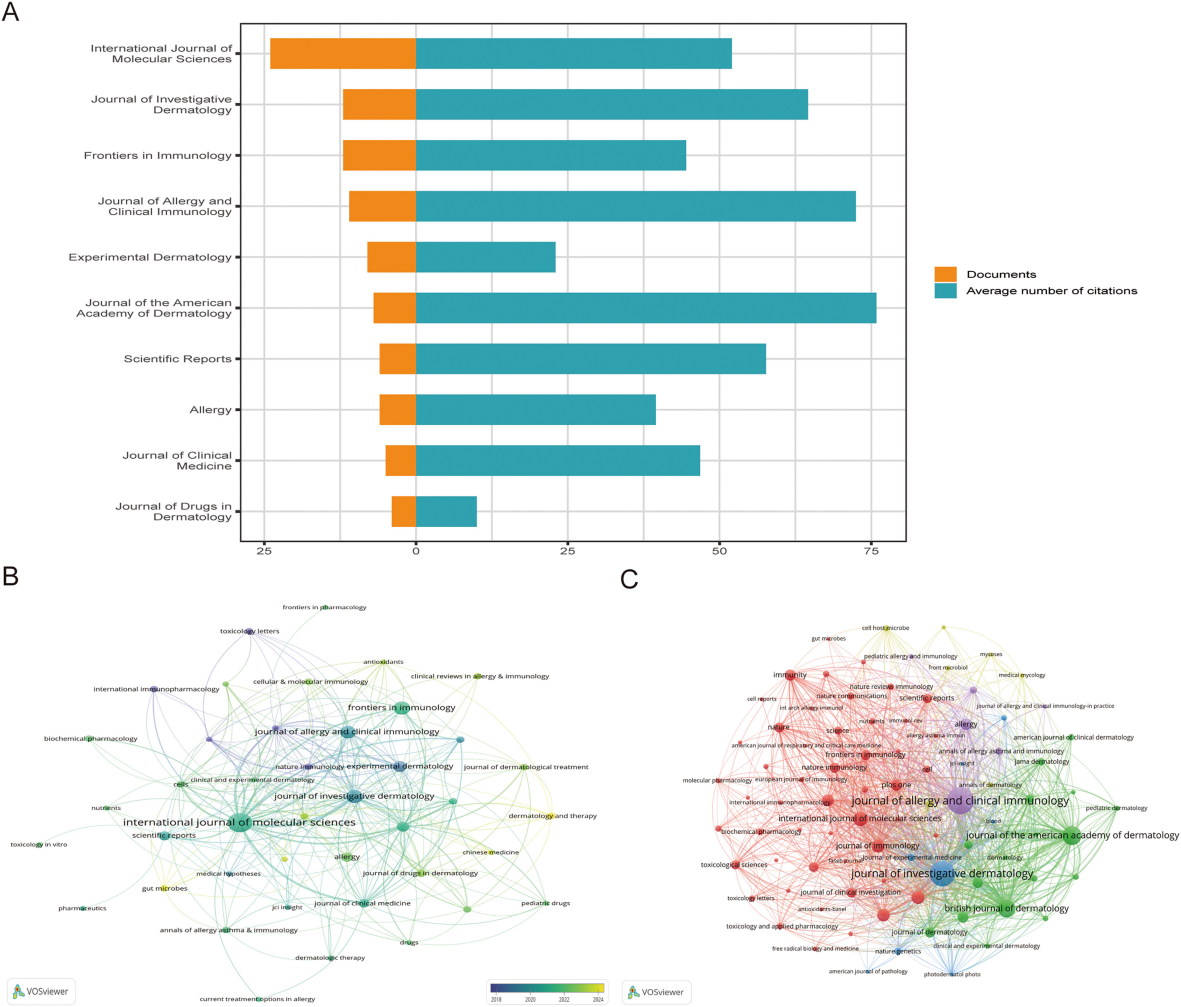
Visual analysis of journals in AD and AHR research: **(A)** journals publishing at least four articles; **(B)** journal citation network; **(C)** journal co-citation network.

A total of 165 journals published research on AD and AHR. Figure 5A presents the journals that published at least four articles in this field. Among them, International Journal of Molecular Sciences had the highest number of publications (*n* = 24), followed by Journal of Investigative Dermatology and Frontiers in Immunology (*n* = 12 each), and Journal of Allergy and Clinical Immunology (*n* = 11). Among the top 10 most productive journals, Journal of the American Academy of Dermatology had the highest average citations per article (75.86 citations/article), followed by Journal of Allergy and Clinical Immunology (72.45 citations/article) and Journal of Investigative Dermatology (64.58 citations/article), indicating that these journals combine both high productivity and strong academic impact.

Figure 5B illustrates the journal citation network, which formed several tightly connected clusters centered on journals in dermatology, allergy, and immunology. Among them, International Journal of Molecular Sciences had the highest total link strength (TLS = 252), occupying a central position in the network. Journal of Investigative Dermatology (TLS = 140), Journal of the American Academy of Dermatology (TLS = 109), Experimental Dermatology (TLS = 68), and Journal of Clinical Medicine (TLS = 65) also showed strong connectivity, indicating that they serve as important hubs for knowledge exchange in this field.

Co-citation analysis (Figure 5C) showed that Journal of Allergy and Clinical Immunology (1,452 citations) and Journal of Investigative Dermatology (1,303 citations) were the two most frequently co-cited journals. The latter also had the highest total link strength (TLS = 134,500), underscoring its central bridging role in the knowledge base of this field. In addition, high-impact general or immunology journals, such as Nature, Immunity, and Journal of Immunology, also occupied prominent positions in the co-citation network, indicating that advances in this field rely not only on core dermatology journals but also on sustained support from fundamental immunology research.

### 3.5 Burst analysis of co-cited references

**FIGURE 6:**
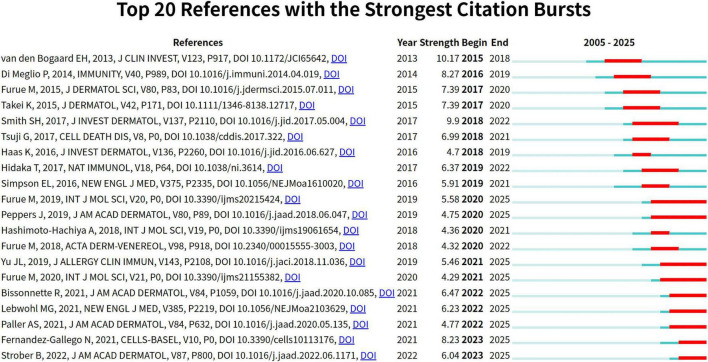
Highly influential co-cited references in AD and AHR research.

Figure 6 presents the 20 co-cited references with the strongest citation bursts from 2005 to 2025. Thematically, these studies can be broadly grouped into three categories: AHR-mediated skin barrier repair and inflammation suppression, mechanistic investigations of AHR in epidermal differentiation and environmental exposure, and clinical evaluations of AHR modulators. Among these references, van den Bogaard et al. (14) showed the strongest citation burst, followed by studies on tapinarof, inflammatory skin diseases, and AHR ligand biology (15–17), indicating that these publications formed highly cited nodes within the co-citation network.

Temporal analysis showed that early citation-burst references primarily focused on AHR-mediated skin barrier repair, inflammatory regulation, and transcriptional control of epidermal barrier proteins (14, 15, 18, 19). Around 2017, burst themes expanded to include the identification of tapinarof, the AHR–OVOL1–FLG axis, keratinocyte-specific AHR function, and air pollution–related AHR activation (9, 16, 20, 21). These shifts suggest that research attention progressively broadened from the interplay between barrier function and immune inflammation to multidimensional topics, including epidermal differentiation, environmental sensing, and neuroimmune regulation.

Since 2019, burst references have further delineated two converging trajectories: diversification of ligand sources and clinical translation, encompassing the AHR/ARNT pathway, microbiota-derived tryptophan metabolites, AHR/NRF2-mediated barrier regulation, AHR ligand biology, and phase 2 and 2b clinical studies of tapinarof for AD (8, 10, 17, 22–24). This burst pattern is consistent with the emergence of keywords such as “tapinarof,” “gut microbiota,” “drug effect,” “drug safety,” and “disease severity” in the keyword evolution analysis. Notably, burst strength reflects the co-citation visibility and citation concentration of the literature within a specific time window, rather than the methodological quality, level of evidence, or clinical superiority of any individual study. The mechanisms and clinical findings of the top 20 references by burst strength are summarized in Supplementary Table 4.

### 3.6 Keyword analysis

**FIGURE 7:**
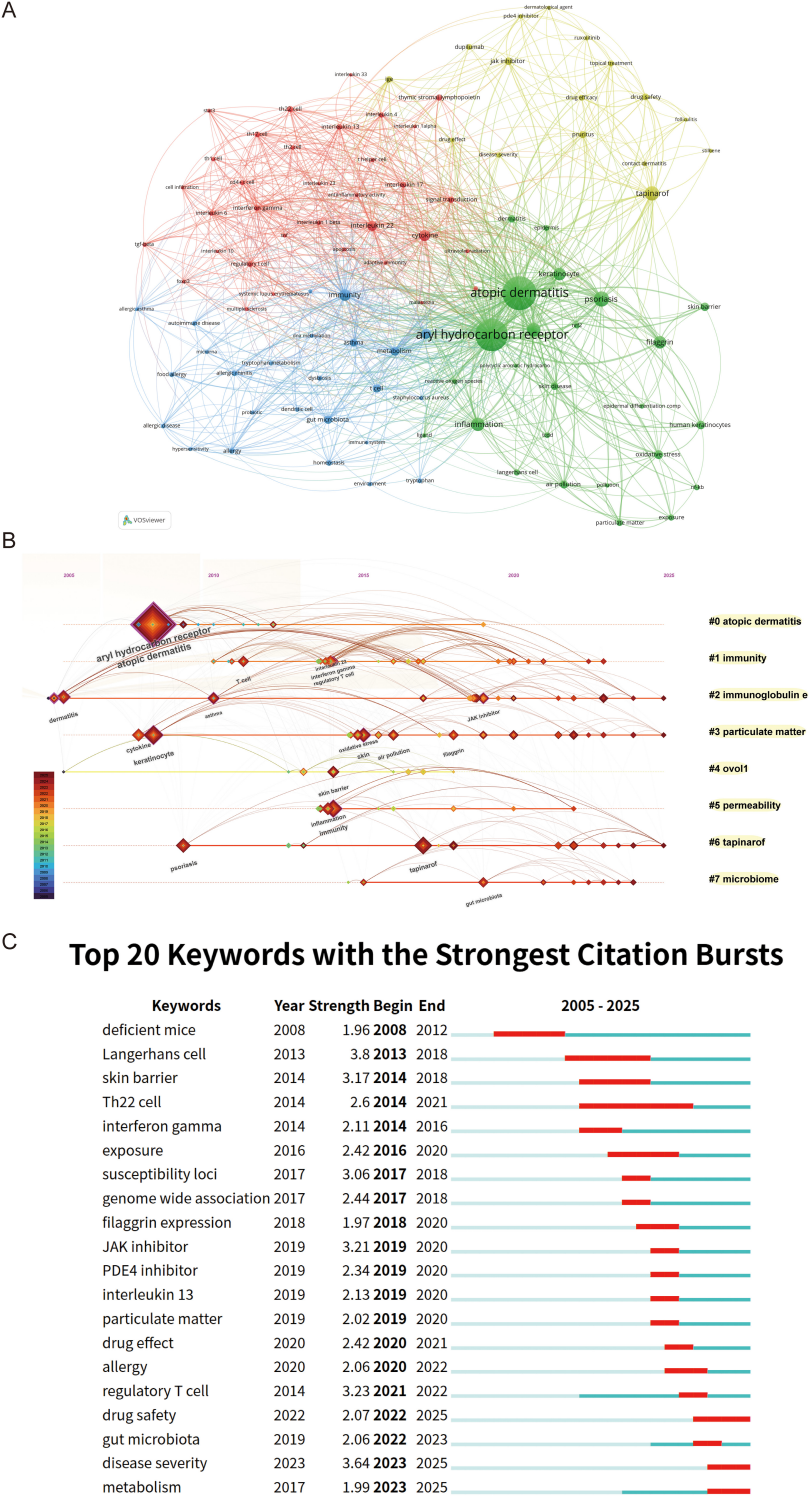
Visual analysis of keywords in AD and AHR research: **(A)** keyword co-occurrence network map; **(B)** keyword clustering timeline map; **(C)** top 20 keywords with the strongest citation bursts.

Visual analysis of the top 100 keywords with frequencies > 7 identified four major clusters (Figure 7A). Atopic dermatitis (*n* = 233, TLS = 1,300) and aryl hydrocarbon receptor (*n* = 217, TLS = 1,267) occupied central positions in the network. The red cluster focused on immune-inflammatory pathways and included cytokine, interleukin 13, interleukin 17, interleukin 22, Th22 cell, regulatory T cell, and thymic stromal lymphopoietin, reflecting imbalances in Th cell subsets and their cytokine networks. The green cluster, represented by keratinocyte, filaggrin, skin barrier, oxidative stress, air pollution, and particulate matter, was associated with keratinocyte differentiation, barrier impairment, and environmental exposure. The blue cluster included gut microbiota, tryptophan metabolism, allergy, asthma, and *Staphylococcus aureus*, highlighting the microbiota–metabolism axis and allergic comorbidities. The yellow cluster centered on tapinarof, JAK inhibitor, PDE4 inhibitor, dupilumab, drug efficacy, drug safety, and disease severity, corresponding to targeted therapies and clinical evaluation. Overall, AHR emerged as a key hub linking four major research directions: immune dysregulation, barrier damage, microbiota-related alterations, and clinical intervention.

The keyword timeline map further illustrated the evolutionary trajectory of this field (Figure 7B). From 2005 to 2025, the main research themes were organized into eight clusters: #0 atopic dermatitis, #1 immunity, #2 immunoglobulin e, #3 particulate matter, #4 ovol1, #5 permeability, #6 tapinarof, and #7 microbiome. In the early stage, high-frequency keywords such as atopic dermatitis, aryl hydrocarbon receptor, dermatitis, cytokine, and keratinocyte predominated, reflecting a focus on the fundamental association between AHR and AD, as well as the inflammatory and epidermal biological basis of the disease. In the intermediate stage, terms including T cell, interleukin 22, interferon gamma, regulatory T cell, skin barrier, inflammation, and immunity emerged sequentially, indicating a shift toward immune dysregulation and barrier dysfunction. In recent years, oxidative stress, air pollution, filaggrin, JAK inhibitor, tapinarof, and gut microbiota have remained highly active, highlighting an expansion of research into oxidative stress, environmental exposure, disrupted barrier homeostasis, and targeted therapy. Overall, the field has evolved from early exploration of basic mechanisms toward broader investigations encompassing immune regulation, skin barrier abnormalities, environmental exposure, and clinical translation.

Burst keyword analysis further corroborated these trends (Figure 7C). The early burst of deficient mice may reflect the foundational role of animal models in establishing mechanistic links between AHR and AD pathophysiology, although citation bursts are also influenced by field-specific citation practices. This was followed by sequential bursts of Langerhans cell, skin barrier, Th22 cell, susceptibility loci, and filaggrin expression, indicating a shift in research focus toward immune-cell regulation, barrier function, and genetic susceptibility. Beginning in 2016, exposure emerged as a new topic of interest. After 2019, JAK inhibitor, PDE4 inhibitor, interleukin 13, particulate matter, and drug effect showed successive bursts, highlighting targeted therapies and environmental exposure as emerging focal points. In recent years, drug safety, gut microbiota, disease severity, and metabolism have continued to exhibit strong bursts, suggesting that current frontiers are centered on the evaluation of efficacy and safety, stratification of disease severity, and regulation of microbiota–metabolism networks. Taken together, the co-occurrence, timeline, and burst analyses delineate a clear evolutionary trajectory in AHR-related AD research: from fundamental immune and epidermal mechanisms, through skin-barrier dysfunction and environmental interactions, to targeted therapy, microbiota-targeted regulation, and refined disease management. This progression also reflects the transformation of AHR from a classical toxicological receptor into a novel therapeutic target in dermatology.

## 4 Discussion

### 4.1 Overall development trends and knowledge structure of AHR research in AD

This study showed that AHR-related research in AD has generally increased over the past two decades, with a marked rise in publications since 2020, suggesting that the field has progressed from an early exploratory phase to a period of steady expansion. The distribution of countries and institutions indicates that the United States, Japan, China, South Korea, and Germany are the principal contributors, with Kyushu University, Dermavant Sciences, Inc., and Northwestern University, as well as scholars such as Furue, M. and Tsuji, G., making particularly notable contributions. However, the collaboration network remains relatively concentrated, and greater cross-national and cross-institutional cooperation is still needed. In terms of knowledge structure, journal co-citation analysis showed that the intellectual foundation of this field relies not only on core dermatology and allergy journals, such as the Journal of Allergy and Clinical Immunology and the Journal of Investigative Dermatology, but also on fundamental immunology journals, including Nature, Immunity, and the Journal of Immunology, highlighting its distinctly interdisciplinary nature. The following discussion focuses on the major research hotspots, evolutionary trends, and future directions in this field.

### 4.2 Research hotspots

#### 4.2.1 Immune-inflammatory dysregulation in the field of AHR and AD

The red cluster of keywords, including cytokine, IL-13, IL-17, IL-22, Th22 cell, regulatory T cell, and TSLP, indicates that immune-inflammatory dysregulation is one of the central research hotspots in studies of AHR in AD. This cluster encompasses Th2/Th17/Th22-associated cytokines, T-cell subsets, and epithelial-derived inflammatory signals, suggesting that this line of research is not confined to a single inflammatory pathway but instead centers on the regulatory role of AHR at the interface between immune-cell responses and keratinocyte-derived inflammatory signaling.

Based on the keyword co-occurrence network and related literature, research within this theme has mainly focused on aberrant AHR expression in immune cells and its association with disease severity (25, 26), the ligand-dependent directionality of AHR-mediated immune effects (27), and AHR-mediated regulation of epithelial-derived inflammatory mediators such as TSLP and IL-33 (28, 29). A recent organotypic skin model study further incorporated AHR into multi-cytokine-driven AD-like epidermal responses, providing additional evidence for its regulatory function (30). This evolutionary trajectory is consistent with the keyword co-occurrence structure, suggesting that AHR research in AD is gradually shifting from immune-cell responses toward epithelial–immune interactions and regulation of the inflammatory network.

#### 4.2.2 AHR in skin barrier homeostasis and environmental exposure

Alongside immune-inflammatory dysregulation, skin barrier homeostasis and responses to environmental exposure constitute another major research focus on AHR in AD. Our keyword co-occurrence analysis identified a green cluster centered on terms such as keratinocyte, filaggrin, skin barrier, oxidative stress, air pollution, and particulate matter, encompassing keratinocyte differentiation, barrier protein expression, oxidative stress, and environmental exposure. In the co-citation burst analysis, early studies on AHR activation by coal tar and Glyteer, the AHR–OVOL1–FLG molecular axis, and keratinocyte-specific gene knockout models constituted the early knowledge base of this research direction. These citation bursts were concentrated in the early phase of the field and primarily centered on AHR-mediated barrier repair, promotion of epidermal differentiation, and antioxidant responses (14, 19–21).

Keyword timeline and burst analyses further showed that this topic subsequently expanded toward environmental exposure. The emergence of the #3 particulate matter cluster, together with burst terms such as exposure and particulate matter, suggests that air pollution and particulate matter exposure have gradually become a recognizable research frontier in this field. This trend is supported by the study by Hidaka et al. (9) on the relationship between air pollution and AHR, as well as by Lee et al. (31) and subsequent PM2.5-related studies (32, 33), and is consistent with the sustained activity of keywords such as “air pollution,” “particulate matter,” and “oxidative stress.” Recent studies on tight junction regulation and AHR–Ovol1–Id1-mediated epidermal–immune homeostasis further indicate that this topic has extended beyond barrier protein expression and epidermal differentiation to encompass the maintenance of barrier architecture and epidermal–immune crosstalk (34, 35).

#### 4.2.3 The emerging microbiota–metabolism–comorbidity axis in AD

Keywords in the blue cluster centered on gut microbiota, tryptophan metabolism, allergy, asthma, and *Staphylococcus aureus*, indicating that AHR-related research in AD has gradually expanded beyond inflammation and barrier dysfunction in local skin lesions to encompass the microbiota–metabolism–allergic comorbidity axis.

Consistent with the keyword timeline and burst analyses, gut microbiota and metabolism have remained active research topics in recent years, suggesting that AHR-mediated barrier maintenance and immune regulation driven by microbiota-derived tryptophan metabolites have emerged as a new research frontier in this field. Multiple lines of evidence support this direction: skin microbiota-derived metabolites such as IAld can modulate keratinocyte inflammatory responses through AHR (10); tryptophan metabolites derived from the gut and skin microbiota participate in barrier protection and immune regulation via the AHR pathway (36–39); and dietary interventions may activate AHR signaling by reshaping the microbiota and its metabolic products (40).

Meanwhile, the co-clustering of *Staphylococcus aureus*, allergy, and asthma reflects the convergence of AHR research with AD-associated dysbiosis and atopic comorbidity networks. On one hand, microbial alterations in AD involve not only excessive *S. aureus* colonization and its effects on barrier lipid metabolism (41), but also the ability of protective commensals to maintain barrier function and suppress pathogens through AHR-dependent or AHR-independent pathways (42–44). On the other hand, epidermal barrier defects that mediate transcutaneous sensitization, together with distal immunomodulation by gut microbial metabolites, are thought to contribute to the atopic march, including food allergy and asthma (45–47). Collectively, this cluster indicates that AHR research in AD has expanded from a local cutaneous pathological node to a broader host–microbe–metabolite–comorbidity network. Given the substantial heterogeneity of current evidence in study models, metabolite detection methods, and clinical endpoints, the practical value of this axis for AD endotyping and intervention remains to be established through standardized mechanistic studies and clinical validation.

### 4.3 Research trends: AHR-targeted therapy and clinical translation

Judging from the evolution of keyword timelines and burst terms, the focus of AHR research in AD has undergone distinct stage-specific shifts: early studies concentrated on establishing the association between AHR and AD, the intermediate phase moved toward elucidating the mechanisms of immune dysregulation and barrier impairment, and recent work has extended in two directions: one emphasizes efficacy evaluation and stratified management centered on actionable targets, and the other explores translational potential represented by the microbiota-tryptophan metabolism axis. The prominent emergence of burst terms such as JAK inhibitors, PDE4 inhibitors, drug safety, disease severity, gut microbiota, and metabolism further indicates that the field is transitioning from mechanistic investigation to a stage in which targeted intervention and microbiota-targeted regulation are advancing in parallel.

In the context of targeted therapy, tapinarof currently represents the clinical translation of AHR research in AD; however, its prominence in the keyword co-occurrence network and keyword timeline map should be interpreted as reflecting research attention and translational activity, rather than evidence of superior clinical efficacy over other topical agents. Clinical reviews and multiphase trials have reported favorable efficacy and tolerability of tapinarof in both adult and pediatric AD (11, 12, 23, 24, 48). The 2025 AAD focused update for adult AD management also issued a strong recommendation for tapinarof cream in adults with moderate-to-severe AD, based on high-certainty short-term evidence (49). Recent reviews have further placed AHR modulators, such as tapinarof, within the broader AD treatment landscape alongside nonsteroidal targeted topical therapies, including topical PDE4 inhibitors and topical JAK inhibitors (50–52). This trend suggests that the therapeutic value of the AHR pathway is being reconsidered within a multi-target, multi-mechanism framework. Clinically, tapinarof may be regarded as a novel AHR-targeted option among nonsteroidal topical therapies, whereas its optimal positioning relative to other topical agents remains to be clarified through further comparative studies.

Meanwhile, the emergence of burst keywords such as gut microbiota and metabolism indicates that another frontier of AHR research is extending toward the gut–skin axis and tryptophan metabolism. Recent reviews have suggested that the gut and skin microbiota, together with their metabolites, may influence barrier function and immune homeostasis through AHR and related signaling pathways (53). These findings point to a potential translational direction in AD, expanding AHR research from mechanisms confined to local skin lesions to microbiota-targeted interventions. However, the precise positioning of AHR modulators within the AD treatment pathway still requires robust evidence on durability of efficacy, safety, and cost-effectiveness across different AD subtypes and diverse populations (54). Similarly, the translational potential of microbiota-targeted interventions requires further clinical validation.

### 4.4 Limitations and future directions

This study has several limitations. First, the literature search was restricted to WoSCC and Scopus. Although combining these databases may improve coverage, relevant studies indexed in other databases, such as PubMed and Embase, may still have been missed. In addition, only English-language publications were included, making language bias difficult to avoid completely. Second, citation counts are influenced by factors such as publication time, database coverage, and self-citation; therefore, highly cited articles do not necessarily represent high-quality evidence. Third, the bibliometric results were used to characterize publication trends, collaboration networks, citation impact, knowledge structures, and thematic evolution in this field; they cannot assess the methodological quality, risk of bias, effect sizes, or certainty of evidence of individual studies. Indicators such as burst references, burst keywords, keyword co-occurrence, and collaboration TLS reflect research attention and academic influence rather than direct mechanistic validation or comparative evidence between therapeutic strategies. The discussion of immune regulation, skin barrier function, the microbiota–metabolism axis, and AHR-targeted therapy in this section represents contextual interpretation based on research hotspots identified by the bibliometric analysis and integrated with existing experimental and clinical evidence, rather than conclusions independently derived from bibliometric analysis alone. Notably, industry-related contributors, including tapinarof and Dermavant Sciences, Inc., were highly visible in the institutional collaboration network, burst references, and keywords. This prominence may reflect academic and clinical interest in AHR-targeted therapy, but it may also be associated with concentrated publication activity driven by industry-led drug development. Moreover, publication-record-based bibliometric indicators cannot distinguish primary reports of pivotal trials from secondary, subgroup, or extension analyses derived from the same parent trial, potentially inflating the apparent volume and bibliometric visibility of industry-sponsored programs. Therefore, such visibility should be interpreted as an indicator of research attention and translational activity, not as comparative evidence of clinical efficacy or therapeutic superiority.

In addition, this study also highlights several gaps in the field. Burst keyword analysis showed that topics such as drug safety, disease severity, and metabolism have emerged prominently in recent years, indicating that research attention is expanding toward long-term safety, disease stratification, and metabolic background; however, the overall evidence in these areas remains at an exploratory stage. Collaboration network analysis further revealed that although the United States, Japan, China, and Germany constitute the major contributors to this field, inter-institutional collaboration remains relatively concentrated, and greater interdisciplinary integration is still needed among basic research, environmental exposure studies, microbiota–metabolism research, and clinical trials.

Based on the above analyses, future research should focus on the following directions: (1) systematically comparing the differential biological effects of AHR ligands from distinct sources, including endogenous ligands, microbiota-derived ligands, environmental pollutants, and drug-derived ligands, on immune regulation and skin barrier homeostasis; (2) integrating studies of the microbiota-metabolite-AHR signaling axis with environmental exposure factors to further elucidate the biological heterogeneity of AD; (3) establishing a clinical evaluation framework that comprehensively accounts for efficacy, safety, disease severity, and long-term outcomes, thereby clarifying the appropriate role of AHR-targeted therapies across different AD subtypes; and (4) strengthening multicenter, multinational basic-clinical collaborative research to accelerate the translation of mechanistic discoveries into precision interventions.

## 5 Conclusion

This study systematically reviewed the current status and evolving trends of AHR-related research in AD from 2005 to 2025 based on the WoSCC and Scopus databases from a bibliometric perspective. The results showed a sustained overall growth in this field, with a particularly rapid expansion since 2020, forming an international research collaboration network led mainly by the United States, Japan, China, South Korea, and Germany. Research topics have evolved from an early focus on the receptor’s role as an environmental sensor to broader investigations of immune and inflammatory regulation, skin barrier homeostasis, and the multidimensional interactions among the microbiome, metabolism, and comorbidities, spanning both basic mechanistic studies and clinical evaluations of targeted therapies.

Despite rapid progress in this field, methodological standardization, long-term safety evaluation, and disease stratification still require further improvement. Future studies should strengthen comparisons of the biological effects of AHR ligands from different sources, integrate multidimensional factors such as the microbiota, metabolism, and environmental exposures, and promote multicenter, multinational collaborative basic and clinical research to facilitate the effective translation of basic discoveries into precision therapy and clinical practice.

## Data Availability

The datasets analyzed in this study are subject to the following licenses/restrictions: the original records were derived from the Web of Science Core Collection and Scopus databases, and access to these records may be subject to subscription and institutional authorization. The processed data supporting the conclusions of this article are available from the corresponding author upon reasonable request. Requests to access these datasets should be directed to YS, songyui1973@163.com.
